# Concrete evidence: outplanted corals for reef restoration do not need extended curing of ordinary Portland cement

**DOI:** 10.1098/rsos.241064

**Published:** 2024-12-04

**Authors:** E. G. Knoester, A. Vos, C. Saru, A. J. Murk, R. Osinga

**Affiliations:** ^1^ Marine Animal Ecology, Wageningen University and Research, PO Box 338, Wageningen 6700 AH, The Netherlands; ^2^ REEFolution Trust, Diani Beach Road 5112-80401, Diani Beach, Kenya

**Keywords:** *Acropora*, artificial reefs, coral gardening, curing concrete, macroalgae, pH

## Abstract

Artificial reefs for coral reef restoration are often concrete-based. After concrete is poured, it initially has a high surface pH (approx. 13), which neutralizes within several weeks. During this curing, colonization by marine microalgae is delayed and also macrobenthos such as corals may be impacted. In this study, we evaluated how concrete curing time applied prior to the deployment of artificial reefs affected coral performance. Fragments of five coral species were outplanted onto ordinary Portland concrete discs (*n* = 10) that had been cured on land. Seven different curing periods were applied, ranging from one day up to four months. The discs with corals were deployed at a Kenyan reef and photographed at the start and end of the experiment. After 1 year, coral cover had increased for four coral species and declined for one, but this was unrelated to concrete curing time. Also, no effect of curing time was seen on the development of other common benthic organisms such as macroalgae or soft corals. We conclude that curing of concrete is unlikely to have any long-term negative impacts on coral performance and therefore, extended curing of artificial reefs prior to coral attachment is unlikely to benefit reef restoration efforts.

## 1. Introduction

Persistent worldwide declines in coral cover have prompted a call for global climate action, improved management of local stressors such as overfishing and pollution, and the upscaling of restoration efforts [1]. Coral reef restoration, as yet, is happening on small and experimental scales, leaving ample room for further optimizations [2]. Typically, coral gardening is applied as reef restoration technique, where corals are first grown in nurseries and then outplanted onto either degraded or artificial reefs [3,4]. Artificial reefs come in many shapes and different materials [5], but concrete is most commonly used [6]. The use of concrete for reef restoration has several benefits, including its durability, stability, safety, availability, shape flexibility and its similar chemical composition to the reef substrate [7]. Indeed, concrete artificial reefs have been effectively used to increase coral and fish abundance, albeit at small scales not exceeding a few hundred square meters [8–10]. Notwithstanding, there are several environmental concerns regarding the use of concrete, including its carbon footprint [11], the unsustainable mining of required resources such as sand [12] and, most directly coupled to reef restoration, the potential negative effects on marine life caused by the high pH of concrete during the first weeks after its production [13].

An initial high pH (approx. 13) of concrete occurs when the aggregate, cement and water are mixed, and the process of curing (i.e. hydration: the hardening of concrete after initial pouring) begins. As concrete hardens during curing and because ingression of carbon dioxide progressively neutralizes the outer layers (i.e. carbonation), the surface pH of concrete typically drops to around 10 within two weeks. Thereafter, the surface pH will continue to decline to reach ambient levels within one to three months, whether cured in seawater [14,15] or freshwater [13,16]. Owing to the higher concentration of carbon dioxide in air, this curing process is typically faster when concrete is cured on land [17,18]. Several short-term (less than one month) studies have shown that when concrete is immediately deployed in the marine environment, the initial high pH can delay microalgal colonization by a couple of weeks [14,17,19–22]. Several longer term studies confirmed this effect on early benthic colonization, but also showed that successional effects did not last as ordinary and pH-neutral concrete developed benthic communities that became indistinguishable within a few months ([15,17,23], but see [24]). This indicates that the effect of initial high pH of concrete might not have any longer term effects on development of macrobenthos such as corals. This is further supported by several studies that show similar benthic communities and hard coral recruitment on concrete and pH-neutral substrates, such as natural rock (e.g. [25,26]). Nonetheless, such comparative studies remain scarce and pH-neutral concrete is still being advocated for increased biodiversity on artificial reefs in general [13,14,20,21,27] and for coral reef restoration specifically (e.g. [28]).

Whether longer curing times and therefore more pH-neutral concrete actually benefits corals remain unknown, as no study to date has evaluated the effects of concrete curing time on hard coral performance. The aim of this study was to address this knowledge gap by determining how curing time of concrete influences coral growth on concrete substrates. Specifically, we explored the potential added value of extended concrete curing times on land before deployment as artificial reefs, so that coral fragments eventually attached to the concrete would be less exposed to high pH values. Coral fragments of five different species were outplanted onto concrete discs that had been cured beforehand ranging from just one day up to four months. Coral growth was monitored for 1 year. Given the initial peak in pH followed by a gradual decrease during concrete curing, we expected lower growth of corals outplanted onto discs with shorter curing times, especially with curing times of less than two weeks.

## 2. Methods

### 2.1. Area description

The study was conducted from June 2021 to June 2022 at Firefly House Reef (−4.6505, 39.3866), a small reef stretch near Shimoni, Kenya. The reef, which is positioned in a kilometre-wide sea strait, is subjected to semi-diurnal tides of up to 4 m difference. Long-term average water temperatures range from a minimum of 25°C in August to a maximum of 29°C in April. Salinity is typically stable at around 35 ppt all year round, but could occasionally drop to around 30 ppt during heavy rains in April and May [29]. The pH in the area fluctuates between 7.8 and 8.2 [30]. About half of the reef substrate is covered by hard corals, with the remainder covered by soft corals, macroalgae, sponges and other sessile invertebrates [31]. Adjacent to the reef there are long stretches of sand interspersed with a mixed assemblage of seagrass, which is where the experiment was set up.

### 2.2. Experimental set-up

On 15 June 2021, a total of 350 concrete discs were deployed on a sandy stretch at a depth of 3 m (low tide). The discs had been subjected to seven different curing times on land beforehand (50 discs per curing treatment): either for one day, three days, one week, two weeks, one month, two months or four months. For example, the discs in the one-day treatment had just been poured on 14 June, the day before deployment, whereas discs of the four-month treatment had already been poured on 15 February. All discs (30 cm diameter, 10 cm height) were made by hand-mixing Ordinary Portland Cement, coral rock aggregate, river sand and fresh water, at a cement/aggregate ratio of 1/3 and a water/cement ratio of 3/5. To allow attachment of an ID label and to facilitate attachment of coral fragments, five tie-wraps were inserted into each disc. Discs were demoulded after 24 h and cured outside in air for the required duration and naturally exposed to the ambient tropical temperatures, rainfall and humidity of the Kenyan coast during the southeast monsoon [32].

**Figure 1.**
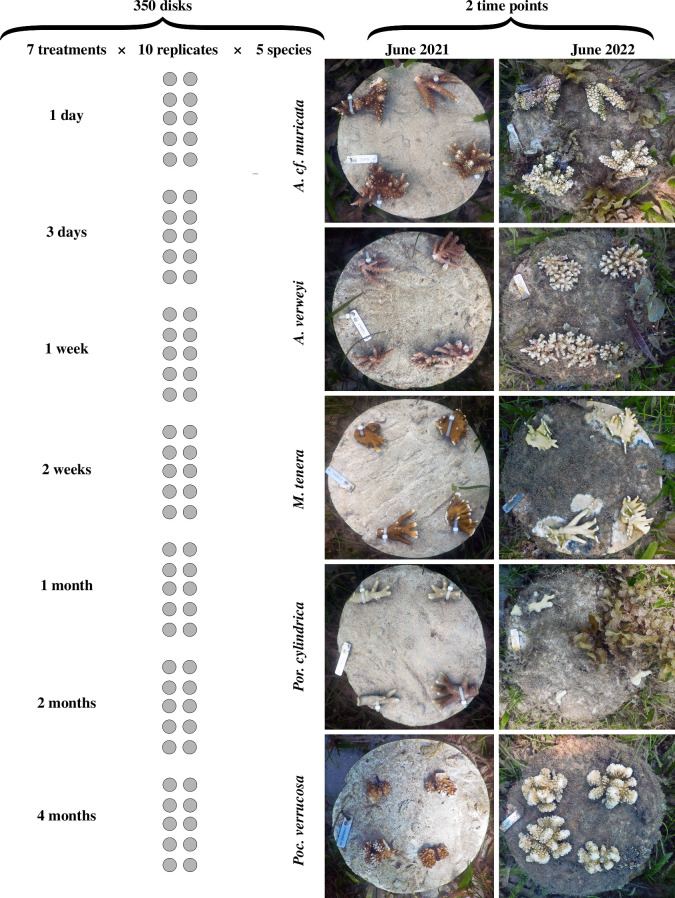
Schematic overview of the experimental set-up. Seven different curing treatments (ranging from one day to four months) were applied to concrete discs, each with four coral fragments attached. The experiment was performed for five different coral species (*Acropora* cf. *muricata*, *Acropora verweyi*, *Millepora tenera*, *Porites cylindrica* and *Pocillopora verrucosa*) and each treatment * species combination was replicated 10 times, totalling to 350 discs. Pictures of each disc were taken at the start of the experiment and again 1 year later to determine the cover of hard coral and other benthic organisms.

Upon deployment, discs of all seven curing treatments were placed simultaneously and arranged in random order in a grid so that all discs were separated by 1 m from each other. Coral fragments (approx. 10 cm length) were harvested and transported underwater from a nearby coral nursery run by the REEFolution Trust, an non-governmental organization focussing on community-managed reef restoration. Five different coral species were chosen for their local abundance and different life-history strategies [33]: two competitive species (*Acropora* cf. *muricata* and *Acropora verweyi*), a generalist species (*Pocillopora verrucosa*), a weedy species (*Porites cylindrica*) and a reef-building hydrozoan (*Millepora tenera*). Each disc was planted with four fragments of a single species. The order of species on different discs was randomized, while ensuring equal distribution of species across the various curing treatments. This resulted in 10 replicate discs for each curing treatment * species combination and a total of 1400 outplanted coral fragments. See figure 1 for an overview of the experimental set-up and sample pictures of the discs and corals. After outplanting of the corals, no further maintenance was performed on the discs.

### 2.3. Monitoring and analysis

The study site was visited monthly to visually evaluate the performance of corals on all discs, noting for example instances of coral predation or bleaching. Pictures of each disc with corals were taken right after deployment and 1 year later. Benthic cover in broad functional groups (i.e. hard coral, soft coral, turf algae of <1 cm, macroalgae of >1 cm and bare substrate) was determined for each picture through manual annotation of 50 random points using *CoralNet* [34]. Only benthic organisms attached to a disc were considered, thus points falling on the surrounding bottom substrate such as sand and seagrass were excluded. No pH measurements were performed before deployment of discs (e.g. by using concrete powder or pore solution), because such internal pH measurements were deemed unrepresentative for what coral fragments would be exposed to externally. No pH measurements were performed after deployment, as the near-endless dilution in seawater would make any external pH measurements uninformative [17]. All analyses were performed in R [35]. To compare hard coral cover over time between the seven different curing treatments and five coral species, a linear mixed-effects model from the *nlme* package was used [36]. In total, 11 discs were either damaged or lost (probably owing to entanglement with fishing gear) and excluded from the analysis. Coral cover was square-root transformed to improve model fit, as evaluated by inspection of residual plots. A Wald *χ*^2^ test from the *car* package [37] was used to determine the significance of the three fixed factors (time, curing treatment and coral species) and their interactions and these were followed up by pairwise comparisons with Tukey adjustments using the *emmeans* package [38]. Change in benthic cover of the other functional groups is presented visually.

## 3. Results

**Figure 2.**
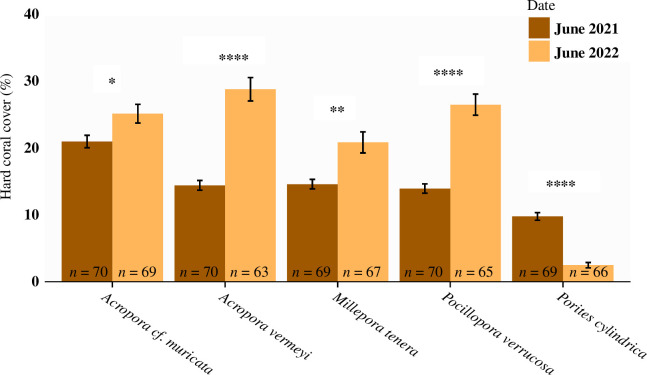
Percentage hard coral cover (mean ± s.e.) between the start and end of the experiment (1 year later) for each of the five coral species (values pooled over curing treatments, replicate numbers are noted at the base of each bar). Asterisks denote significant differences in coral cover between the two time points for each species (*p < 0.05, **p < 0.01, ***p < 0.001, ****p < 0.0001).

Towards the end of the experiment (April 2022), water temperatures shortly peaked above the bleaching threshold of 30°C, resulting in a maximum temperature anomaly of 3° heating weeks [39]. While all coral species paled throughout this period (see also figure 1), no instances of full bleaching were encountered. Overall, corals remained well attached and grew throughout the experiment. However, species-specific differences were observed (figure 2) and confirmed by a significant interaction between time and coral species in the ANOVA ($\chi_{4}^{2}$ = 264.11, *p* < 0.001). *Acropora verweyi* grew rapidly (increasing its cover from 14% to 29% in 1 year), whereas moderate growth was seen for *Poc. verrucosa* (14% to 26%), *M. tenera* (14% to 21%) and *A*. cf. *muricata* (21% to 25%). Interestingly, growth by *M. tenera* was predominantly achieved by encrusting over the concrete disc, rather than by branch growth that was seen for the other species (see figure 1). For both *Acropora* and *Pocillopora*, branch extension at the edge of the discs was frequently stunted owing to abrasion by seagrass. Cover of *Por. cylindrica* showed a completely different trajectory than the other species and decreased throughout the experiment from 10% to 2%. Broken pieces of *Por. cylindrica* were often found, typically still alive, hinting at coral predation by fishes. Throughout the 1 year study, only a handful (<5) discs suffered from predation by invertebrates, which was consistently caused by *Drupella* sp. snails partially predating on outplanted *Acropora* spp. Generally, a thin layer of sand covered most discs and no natural recruitment of hard corals was observed on any disc in this seagrass habitat.

**Figure 3.**
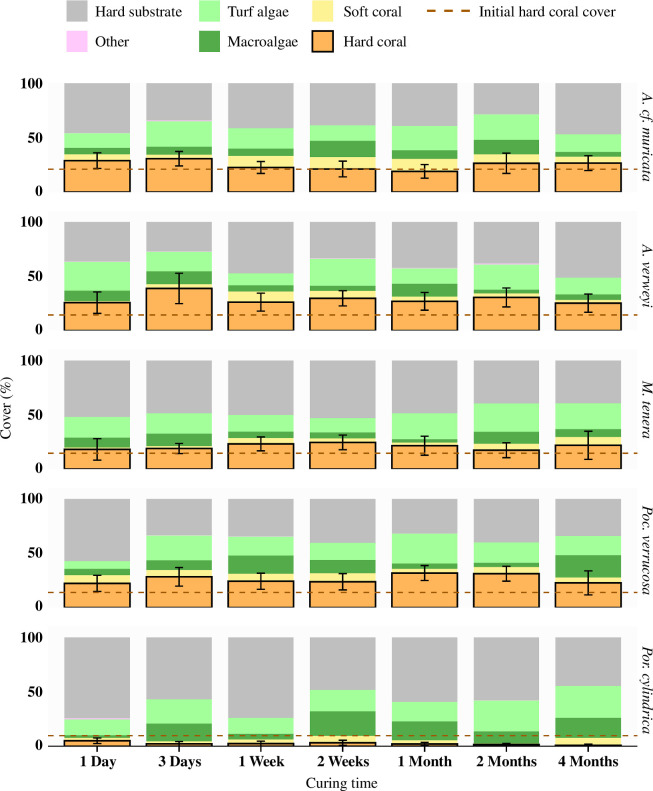
Benthic cover at the end of the 1 year experiment, split per species (*Acropora cf. muricata*, *Acropora verweyi*, *Millepora tenera*, *Pocillopora verrucosa* and *Porites cylindrica*) and curing treatment (*n* = 7–10). Average cover of hard coral is outlined in black with 95% confidence interval noted by the error bars. No significant differences in hard coral cover between curing treatments were found for any of the species. Average hard coral cover across all curing time treatments at the start of the experiment is indicated by the dark red line for each species. Other benthic categories include hard substrate (bare concrete), turf algae (<1 cm), macroalgae (fleshy algae >1 cm), soft coral and a pooled category ‘other’ that includes rare sessile invertebrates such as sponges, tunicates and hydroids.

Crucially, no effect of concrete curing treatment on coral cover was observed, and this was consistent for all five species studied (figure 3). Consequently, no significant difference in coral cover was found between the seven curing treatments ($\chi_{6}^{2}$ = 8.09, *p* = 0.231), nor were any significant interactions found between treatments and coral species ($\chi_{24}^{2}$ = 28.90, *p* = 0.224), treatments and time (*$\chi_{6}^{2}$* = 7.56, d.f. = 6, *p* = 0.272) or the combination of all three factors ($\chi_{24}^{2}$ = 18.02, *p* = 0.802). Thus, coral cover of the studied species increased or declined regardless of the curing treatment of the concrete substrate onto which the fragments were attached. Similarly, no clear pattern across curing treatments could be observed for any of the other three functional groups (figure 3). In general, macroalgae (mainly *Sargassum* spp. and *Padina* spp.) and soft corals (typically Xenidae) were present in small numbers (average across all discs 9% and 5%, respectively), whereas turf algae (19%) occupied an area that was roughly equal to the average area covered by the hard corals (21%). The remainder of the discs remained mostly bare concrete (45%, see also figure 1).

## 4. Discussion

This study aimed to determine whether extending the curing duration of artificial reefs on land before deployment in the ocean could benefit coral performance, supposedly by reducing the exposure of outplanted corals to the initial high pH of uncured concrete. In contrast to our expectations, the duration of concrete curing did not influence coral performance in this 1 year study. Most corals grew well, even when attached to concrete that had been poured just a day earlier. While coral growth varied among the five studied species, the absence of a curing time effect was consistent. In addition, no effect of the different curing times of concrete was observed on other benthic organisms such as macroalgae and soft corals. Probably, the near endless dilution in seawater prevents any consequential effects of curing duration on macrobenthos, especially in the longer term. For reef restoration, this implies that extended curing periods or the use of pH-neutral concrete do not confer any readily observable benefits to longer term performance of outplanted corals.

To evaluate the wider applicability of these results, four aspects of this study are placed in context. First, pH of concrete may vary depending on the exact ingredients, mixing ratios and curing conditions used [20]. This study applied the commonly used ordinary Portland cement without any additives and is therefore probably representative for many reef restoration projects worldwide [6]. However, an *in situ* quantification of concrete’s surface pH could help to further verify this. Second, the local environment can modify outcomes. For example, an effect of curing duration might possibly be detectable in areas with only limited water exchange. Reef restoration projects, however, typically occur in well-flushed areas like this study location, as these conditions are beneficial for coral development [40]. Third, it is conceivable that smaller fragments are more sensitive to any effect of curing concrete. However, the size used in this study is typical for restoration projects [40], precisely because larger fragments can better withstand potential stressors [41]. High concrete pH may initially have a greater impact on small fragments, but, as with microalgae [17,23], any potential effect is expected to be short-lived as the alkaline compounds will swiftly be washed out and is therefore probably irrelevant for the longer term reef restoration success. Coral recruits have been shown to settle well on concrete artificial reefs [8] and it would be worthwhile to evaluate how artificial reef placement can be best timed to maximize such natural recruitment. Finally, the performance of some outplanted coral fragments in this study was hindered by several biotic and abiotic factors, including predation, abrasion, sedimentation and bleaching. The effect of shorter curing did not make the corals more susceptible to additional stressors. Clearly, other factors than the initial high pH of concrete are more relevant for coral performance and reef restoration, such as site-specific conditions [23,25], habitat structure [17], topographical complexity [27,42], substrate orientation [43], sedimentation rates [26], substrate durability [44] and ecological interactions like herbivory [45] and coral predation [31].

Our findings challenge the majority of studies that reported negative effects of concrete’s high pH on benthic organisms [14,17,19–22]. However, many of these studies were performed over extremely short timespans (less than one month) for reef development and focused on small and pioneering organisms such as microalgae, instead of macrobenthos such as hard corals. Interestingly, several of these studies reported positive effects of curing concrete on alkali-tolerant organisms such as barnacles and oysters [14,20,21]. As corals need a high (internal) pH to form their skeleton, one might even postulate that curing concrete could have positive effects on corals [25], especially in conditions of progressive ocean acidification. Clearly, the short-term negative effects of high pH on microalgae as found in prior short-term studies cannot simply be extrapolated to coral performance on timescales relevant for reef restoration. Based on the absence of any effect of concrete on macrobenthos in the few longer term studies [17,23] and the lack of effects on corals in this study, we conclude there is no indication of negative effects of initial high pH on the performance of corals attached to concrete structures. It remains unknown, however, whether concrete is the optimal substrate for reef restoration and comparisons of coral performance on various artificial reef materials and the natural reef substrate remain an important research direction [46].

Furthermore, concrete does pose environmental concerns that should be considered when developing reef restoration plans. First, as climate change is currently recognized as the biggest threat to coral reefs [47], reductions in carbon footprint are needed. A promising opportunity is the replacement of cement with green alternatives or industry by-products such as blast furnace slag, which could reduce the carbon footprint by over 90% at competitive costs [11]. Potential effects of leaching metals should be considered, although these might be minimal owing to the reduced permeability of concrete with such additives [48]. Second, as resources such as sand are limited, recycling of aggregate ingredients should also be advocated. For example, up to 98% of the filler of a concrete mix can be sourced from industrial wastes, lowering the costs without compromising quality [21]. In addition, the use of seawater instead of scarce fresh water to mix concrete appears to be a viable option for artificial reefs, as these typically lack rebar reinforcement and therefore have no corrosion risk [13,14,16].

We conclude that extended curing time of concrete is not needed to improve the performance of outplanted coral fragments for reef restoration. As the expected spike in pH of curing concrete had no visible long-term effects on corals, we argue that a more effective avenue to achieve coral reef restoration and conservation benefits would be to focus on exploring concrete ingredients and alternatives that can achieve a reduction in carbon footprint or reduce the reliance on scarce materials.

## Data Availability

Data and relevant code for this research work are stored in GitHub [49] and have been archived within the Zenodo repository [50].
